# The effect of legislation on the treatment practices and role of naturopaths in South Africa

**DOI:** 10.1186/s12906-020-02916-5

**Published:** 2020-05-06

**Authors:** Wendy Ericksen-Pereira, Nicolette V. Roman, Rina Swart

**Affiliations:** Faculty of Community Health Sciences, University of the Western Cape, Bellville Medical Campus, Blankenberg Road, Bellville, Cape Town, 7535 South Africa

**Keywords:** Complementary medicine, Regulatory body, Scope of practice, Treatment practices, Legislation

## Abstract

**Background:**

In South Africa naturopaths have been practising for over half a century. Over this period, changes in legislation have resulted in different levels of training and registration processes - which has impacted on the profession in various ways. This paper explores the effect of legislation on the treatment practices and role of naturopaths in South Africa.

**Methods:**

This was a qualitative study which used an exploratory approach. Participants were sampled from the list of naturopaths registered with the Allied Health Professions Council of South Africa (AHPCSA). A set of 15 open-ended survey questions were emailed to 59 naturopaths. Twenty one naturopaths participated: 13 responded via email and eight were interviewed. Responses were coded and thematically analysed.

**Results:**

It was found that despite differences in training and years of practice experience, four core treatment practices of diet therapy, lifestyle medicine, supplementation and physical therapies were common to all participants with the older, more experienced naturopaths using a wider range of treatment practices. There is a shared common vision of wanting the profession to have greater participation in the public healthcare system. This research has found that legislation influences the treatment practices and role played by naturopaths in South Africa. The findings of this paper acknowledges the limiting impact of state legislation on naturopathic and other complementary medicine professions.

**Conclusion:**

Naturopathy has to operate within the legislative framework and this appears to be one of the key factors which has contributed to the lack of growth of naturopathy in South Africa. Findings thus highlight the need for new legislation to reflect the changes in society to ensure that the emergent healthcare needs of the population are met.

## Background

The World health Organisation (WHO) defines complementary medicine as those healthcare practices which are not part of a country’s traditional or conventional allopathic healthcare system [1]. Naturopathy is recognised as a system of complementary medicine (CM) [2]. Naturopathic medicine is underpinned by the Aristotelian [3] and Hippocratic [4] philosophy of vitalism and wholism. The principles which guide naturopathic practice have their roots in this philosophy and were codified as: the healing power of nature, first do no harm, treating the root cause of an illness, treating the patient wholistically, the role of the naturopath as teacher, and the prevention of illness and disease [4] It is this philosophy and foundational principles which guide the assessment, diagnosis and treatment practices of the naturopath [5, 6].

### Naturopathy in South Africa

The history of naturopathy is intertwined with the history of CM in South Africa until 1985 when legislative changes resulted in the recognition and growth of the chiropractic and homeopathic professions.

CM, in the form of homeopathy, was first introduced into South Africa by European immigrants and was practised in the country since the 1800s [7]. After the second world war South Africa experienced a wave of European immigrants, among them homeopaths, naturopaths and herbalists [8]. The 1960s and early 1970s saw a proliferation of private training colleges for naturopathic, homeopathic, chiropractic and osteopathic medicine. One of the first professional organisations for these professions was the South African Naturopathic and Homeopathic Association, founded in the 1960s by Dr. William Lilley [7].

With the passing of the Homeopaths, Naturopaths, Osteopaths and Herbalists Act 52 in 1974 [9], all CM practitioners recognised by the act were expected to register within 6 months from the date of promulgation of the Act with the Department of Health. Many naturopaths who registered as a result of this Act were trained in more than one diagnostic profession (a profession legally permitted to diagnose and treat patients [8]) through private colleges which existed at the time. As a result, many naturopaths applied for dual registration as naturopaths and homeopaths or osteopaths. This register was closed to practitioners in 1975. However, students at private training colleges at the time were allowed to complete their studies and register [8]. Training colleges were gradually phased out and by 1982 all training colleges were closed. The Associated Health Service Professions Act 63 of 1982 [10] resulted in all the registers being closed and no new registrations were allowed. This made it illegal to practise naturopathy, herbalism, homeopathy, chiropractic and osteopathy if unregistered.

The objective of this act was to protect and promote the public health, and control registration and practice of the different CM professions (Act 63 Section 2(1)3) [10]. The act also made provision for the establishment of the South African Associated Health Service Professions Board. As a result of the work of this board the scope of practice (SOP) for the five recognised professions was developed and published in the Government Notice Regulation 2610 in 1982 [11]. In 1985 the register was opened to chiropractors and homeopaths and shortly thereafter training programmes in these professions were established at tertiary institutions [7]. The passing of the Chiropractors, Homoeopaths and Allied Health Service Professions Amendment Act 40 (South Africa) 1995 No. 16643 [12] allowed practitioners who had trained in the 1970s but missed the cut-off date for registration as well as those who trained abroad after 1975 to apply for registration. Also included were individuals who completed various complementary medicine courses at unregistered training centres in the country and practiced without registration. All categories of applicants were expected to undergo 2 years training to upgrade their knowledge and skills and complete a council regulated examination (CRE). Those who passed were registered. This was called the ‘grandfather’ clause.

In 1997, naturopaths formed a professional association, the South African Naturopathic Association (SANA) and joined with the Confederation of Complementary Health Associations of South Africa (COCHASA) to lobby for changes in legislation [13]. Regulation 127 of 2001 [14] meant certain sections of Act 63 of 1982 [10] were amended, which allowed for the registration of ayurveda, chinese medicine and acupuncture, naturopathy, osteopathy and phytotherapy professions. This allowed practitioners who had not been ‘grandfathered’, or had studied either locally or abroad and could provide documentary evidence of training, to apply to write a CRE [7]. The regulation also set out educational training requirements, paving the way for training of naturopaths and other legally recognised CM professions at tertiary level. This led to the establishment of the only training programme for naturopaths in the Faculty of Community Health Sciences at one of the established universities, which saw the first cohort of naturopaths graduate in 2006 [8].

Although sections of Act 63 were repealed and new regulations introduced, all legislation should be read in conjunction with Act 63 [10] as collectively they set the parameters for legal practice.

### Public awareness of naturopathy

Unlike other fields of medicine, there is little public awareness of the naturopathic profession, even though the latter has been in practice for over 50 years in South Africa. Responsibility for promoting a profession to the public lies with the professional associations [15]. This requires a clear understanding of the broader healthcare system, the regulations which govern the profession and treatment practices which naturopaths may use. This allows the professional body to promote the profession to the broader public [16], by providing a framework from which to better understand the nature and practice of the profession. Research documenting the effect of legislation on the role and treatment practices of naturopaths has, until recently, been lacking. This paper aims to describe the effect of legislation on the role and treatment practices of naturopaths in South Africa, as perceived by registered South African naturopaths.

## Method

Ethical approval for the study was obtained from the University of the Western Cape’s Senate Ethics Committee. All information gathered is securely kept and accessible only to the researcher. Signed informed consent forms were obtained from all participants.

In this qualitative study an interpretive research paradigm was used, as interpretivism assumes “that reality should be interpreted through the meaning that research participants give to their word” (p.309) [17]. This study used an exploratory approach, as there is a lack of information on this topic [17] in South Africa. Participants were sourced from the website of the Allied Health Professions Council of South Africa (AHPCSA) [18], the regulatory body with whom all diagnostic CM practitioners are registered, as required by Act 63 of 1982 [10].

Names of registered naturopaths were identified from the register. Due to legal requirements, AHPCSA members’ contact details were sought through an electronic search of the white pages telephone directory, medical practitioner sites, Facebook and Google, which yielded details for 64 AHPCSA registered naturopaths. These naturopaths were then contacted telephonically or via email. The purpose of the research was explained, and they were invited to participate in the study.

Five of the naturopaths who were registered prior to the passing of Regulation 127 of 2001 [14], refused to participate, eight agreed to be interviewed, and the remainder asked for the list of questions to be forwarded via email. A total of 59 naturopaths were sent a set of 15 open-ended survey questions, an ethics clearance and an informed consent form via email. Examples of the open-ended questions were [1]: Provide a definition of what you feel a naturopath is [2]; Describe the various therapeutic treatment practices you use with your patients [3]; Discuss the role that you feel naturopaths play in the South African healthcare system [4]; How would you like to see this role in the future?

Thirteen naturopaths forwarded responses to the questions via email. Interviews with eight naturopaths were conducted at a time and venue convenient to participants. Questions posed in the interviews were the same as those sent via email. Interviews were recorded and transcribed verbatim. Once all data was collected, participants responses were coded in order to ensure anonymity. All responses were summarized according to the questions and then thematically analysed [19].

## Results

Twenty one naturopaths agreed to participate. Practice experience of participants ranged from newly qualified to over 50 years of experience. Table 1 summarizes the practice experience of participants.

**Table 1:** Participants practice experience

Registered naturopaths	Over 50 years	Between 40 and 49 years	Between 30 and 39 years	Between 20 and 29 years	Between 10 and 19 years	Between 5 and 9 years	Between 0 and 4 years
**No of years since completion of naturopathic studies**	**1**	**1**	**1**	**2**	**3**	**10**	**3**
**Full time practice**	**1**	**1**	**1**	**2**	**3**	**3**	**1**
**Part-time practice**					**0**	**3**	**1**
**Not in practice at all**					**0**	**1**	**1**
**Postgraduate studies**					**0**	**3**	**0**
**Dual registration**	**1**	**1**	**1**	**0**	**0**	**0**	**0**

The eight most experienced naturopaths were the same participants who agreed to participate only by being interviewed. They all requested more information about the researcher and the project compared to less experienced naturopaths.

The ethics clearance document was explained and credentials of the researcher as a registered naturopath were established. Participants acknowledged that they preferred speaking to the researcher as they were weary of divulging information on naturopathy when they did not know how the information would be used. Naturopaths who were graduates of the tertiary training programme were willing to answer questions electronically and email their response.

Participants’ naturopathic training ranged from unregistered private colleges in the 1960s to the 1990’s, naturopathic training colleges abroad and graduates of the AHPCSA accredited tertiary training programmes. Table 2 summarizes the levels of training of participants.

**Table 2:** Participants training

Training institution	Trained at unregistered naturopathic colleges in South Africa	Trained at Lindlhar college in South Africa	Trained at naturopathic colleges abroad	Trained in South Africa at the tertiary training institution.
**Number of participants**	6	2	2	11

When asked about their training, older, more experienced naturopaths went into detail explaining how they were trained. They viewed themselves as early pioneers of naturopathy in South Africa and spoke with a sense of pride about the diverse treatment practices they were trained in and used in practice. There was also a sense of pride in having been able to practice for the length of time which they had - despite changes in regulations which they felt had a negative effect on growth of the profession. Two naturopaths admitted they had practiced ‘illegally’, as unregistered health practitioners for many years, until legislation changed in 1995 [8]. From the responses of some older naturopaths it would appear the introduction of legislation in 1974 [9] led to a period of strict punitive enforcement of the law which had left its mark on them and could explain why five naturopaths refused to participate (see below: 2).

Practice experience of participants in this study ranged from the first group of naturopaths who trained in South Africa in the 1960’s to the most recent naturopathy graduate. Data obtained from this research indicated that all the naturopaths with more than 10 years practice experience were in full-time practice whereas only four naturopaths with less than 10 years of experience are in full-time practice. Respondents in part-time practice expressed the wish to work as full-time naturopaths. Five were in part-time practice for a while but found it a financial challenge and gave up their practices.

Survey questions and interview responses were analysed thematically and the following themes were identified:

1. Definition of naturopathy
2. Naturopathic registration
3. Treatment practices
4. The role of naturopaths in South Africa: integration into the public healthcare system

### Definition of naturopathy

In South Africa Regulation 127 of 2001 [14] defines naturopathy as: “a system of healing based on promoting health and treating disease using the body’s inherent biological healing mechanisms to self-heal through the application of non-toxic methods.” This definition does not encompass other naturopathic principles such as treating the root cause of disease, treating the patient wholistically and the role of doctor as teacher [20].

The older respondents provided a more general definition of the term which was closer to the legislated definition of naturopathy. This could be due to some of them having been involved in lobbying for changing the legislation which led to the opening of the register to naturopaths in 2001 [13]. For example:

“Naturopathy is a system of healing that uses natural methods and remedies to support and enhance the body’s natural healing mechanisms in order to treat disease and promote health and well-being” (participant 8).

More recent naturopathic graduates incorporated more of the principles of naturopathy into the definition of naturopathy – this could be attributed to the difference in training, the progression and refinement of naturopathic philosophy, terms and practices over the years and the increased availability of naturopathic literature, compared to the 1960’s and 70’s.

“It is a distinct, comprehensive, patient-centric system of medicine which focuses on the root cause of disease, and takes into account the ‘whole’ person by addressing physical, mental, emotional, social, environmental, spiritual and genetic factors” (participant 11).

All participants identified themselves as naturopaths, regardless of whether they were in practice or not, based on the principles which underpin naturopathy.

“I will always be a naturopath as the basic underlying principles of naturopathy you can take into any area of life or work. I am currently working in the public health system where we do a form of analysis called root cause analysis to uncover the root cause of the problem in the health system. This is what we as naturopaths do - unpack the root cause of a patient’s disease” (participant 16).

Naturopaths who are not in practice continue to identify as naturopaths as they are still on the naturopathic register [18] and legally have the title of naturopath. Since they are not in practice, they continue to apply the principles of naturopathy to their everyday lives and in the workplace.

### Naturopathic registration

Respondents’ work experience could be categorized as either: full-time practice, pursuing postgraduate qualifications, part-time practice or not in practice at all (see Table 1). Registration with the regulatory body was important to all participants since it allowed them to use the title of naturopath and to practice legally. Although all respondents were registered with the AHPCSA, for some respondents this had not always been the case as some had previously been denied the opportunity to register due to legislation:

“I completed my training in the 1980’s but wasn’t allowed to register. I practised as a natural medicine practitioner even though I trained as a naturopath. With the new law introduced in 1995 I was ‘grandfathered’ and passed the exam. I could register and use the title naturopath for the first time” (participant 3).

“Naturopaths who didn’t register in the six month window period either went underground and used creative titles to continue practising or were forced to stop practising” (participant 2).

“I was arrested at my practice in front of my patients in the 1970’s because I practiced without being registered. It cost me everything to defend myself. After I got off on a technicality I was constantly watched” (participant 1).

Participants identified some of the challenges with registration. One of the participants reported the following experience when they failed to pay their registration fees:

“I didn’t pay my fees as I had gone back to studying. I was deregistered. There was so much bureaucracy and all the outstanding fees that had to be paid … … .it is easier to pay your fees than go through that process” (participant 18).

If registration lapses due to lack of fee payment, the practitioner is not only deregistered, but required to pay an application fee and double or triple the annual fee, if they wish to be registered again [21]. Conditions for registration are set in out in Regulation 127 of 2001 [14]. Students are required to register within the first 6 months of graduating or undergo a competency assessment (for which they have to pay). The challenge around registration for naturopathy graduates appears to be the cost of registration fees - currently approximately 20% of the average salary of a new graduate [22].

Those who were not in practice indicated that they maintained their registration because they hoped to go into full-time practice when they were in a financial position to do so. Having completed a 5 year naturopathy training programme at a university, it is the general wish of graduates to enter practice. This poses a challenge, as they may not have the funds to go into full time practice immediately after graduating – however, they still have to pay the full annual registration fee.

“After graduating my main source of income came from working in a health shop, after a few months I started a practice part-time” (participant 3).

Only once naturopaths are legally registered can they practice. Without being registered, the title naturopath may not legally be used. The title of naturopath is protected in South Africa as Act 63 of 1982 [10] sets out the legal definition of the term naturopath: “a person registered as such under this Act.” For older naturopaths, registration was a source of pride and achievement, for they were able to overcome the challenges to registration, while for more recent naturopathic graduates, registration also provided a sense of achievement, at having completed a 5 year training programme:

“I was involved in COCHASA - lobbying the government to amend Regulation 63 so that the register could be opened to naturopathy and other CM professions. We presented our case to the Parliamentary Portfolio Committee on Health in November 2000 and the committee voted for the opening of the registers. Finally I and other naturopaths could register” (participant 2).

### Treatment practices

Respondents reported using a range of treatment practices, summarised in Table 3:

**Table 3:** Reported treatment practices

Type of treatment	Proportion of practitioners practising treatment
Diet therapy	100%
Lifestyle medicine	94%
Supplementation	94%
Physical therapies	88%
Botanical medicine	69%
Acupressure	25%
Acupuncture	25%
Homeopathy	25%
Iridology	25%
Exercise therapy	20%
Hydrotherapy	20%

These results indicate the variety of treatment practices used by naturopaths. Of the 11 treatment practices identified, four were used by all respondents. These treatment practices focussed on diet therapy, lifestyle medicine, supplementation and physical therapies. Naturopaths who used a more extensive range of treatment practices were older naturopaths, and either hold dual registration as a naturopath and homeopath, or trained abroad:

“I use a wide range of treatments, depending on what my patients need: lifestyle counselling, diet therapy, homeopathy, herbal medicine, hydrotherapy, massage therapy and iridology” (participant 4).

Less experienced naturopaths who trained in South Africa used a limited number of treatment practices, which reflects on the training received:

“Due to our limited scope of practice I only used diet therapy, herbal therapy, vitamin supplementation, lifestyle therapy and massage therapy when I was in practice” (participant 16).

Regardless of training, the results indicate all participants used treatment practices which are within the naturopathic scope of practice (SOP) as set out in Act 63 of 1982 [10].

### The wider role of naturopaths in South Africa

South Africa has a system of dual healthcare: one public and one private. All participants expressed the wish to see naturopaths play a more significant role in the healthcare system, expressing the hope that naturopathy be incorporated into the public healthcare system. Their reasons for this varied. It was felt that there is insufficient public awareness of the profession of naturopathy - what it is and services naturopaths offer - and that the professional associations do not adequately promote the profession to the public:

“They should play a much bigger role. They are not doing enough to promote naturopathy in society … .We need more people to be exposed to naturopathy and what we do” (participant 14).

This, along with the current legislation, is seen as curtailing naturopaths’ ability to set up a viable practice. Regulation 127 of 2001 [14] sets out the few remedies (emollients, tissue salts, vitamins and minerals) which naturopaths may use in practice listed in Table 4.

**Table 4:** Remedies naturopaths may prescribe

Remedy	Explanation
Vitamins	Excludes injectables and other substances containing an injectable form of Vitamin A or B12
Emollients	Substances that are exclusively intended for application to the skin which are not scheduled or prepared according to homeopathic principles
Minerals	Any mineral that is not scheduled or prepared according to homeopathic principles
Scheussler’s Tissue Salts	Calcarea flouorica; Calcarea phosphorica; Calcarea sulphuricum; Ferrum phosphoricum; Kali muriaticum; Kali phosphoricum; Kali sulphuricum; Magnesia phosphorica; Natruim muriaticum; Natrium phosphoricum;Naturium sulphuricum; Silicae. The above substances to be in one part per million.

Regulation also prevents naturopaths from working in interdisciplinary practices with registered Health Professions Council of South Africa (HPCSA) practitioners. Section 8A of Board Notice 26 published in Government Gazette 36,183 of 2013 states: “A practitioner shall not share his or her rooms with a person or entity not registered in terms of the Act” [23]. This regulation forces naturopaths and other AHPCSA registered practitioners to work within the private healthcare system.

“ … it is very restrictive to naturopathy as a profession due to the limited access to remedies, making the profession less viable financially. We are also unable to work in an interdisciplinary practice with medical doctors. This discourages students from joining the profession and encourages practitioners to leave the country or abandon the profession or practice only part time, whilst they take on other jobs in order to pay the bills” (participant 21).

As a result, practitioners struggle to establish viable practices. It is believed that by becoming part of the public healthcare system, the public will be more exposed to naturopathy and demand for the services of naturopaths will increase. As a result of this increased demand, there would also be a greater interest in studying naturopathy.

Participants felt that the underlying philosophy which guides naturopathic practice, with its emphasis on health promotion and disease prevention, means naturopaths could contribute to the prevention of chronic illness and diseases of lifestyle, thereby lessening the burden of the high cost of non-communicable diseases on the healthcare system.

“Naturopathy can be immensely valuable in the primary healthcare setting in helping to prevent chronic illness and lessen the burden on the (public) healthcare system. Unfortunately naturopathy can only provide a service within the private healthcare system.” (participant 9).

## Discussion

The roles and practices of naturopaths have been extensively shaped by the historic-legal context in South Africa. Therefore this discussion aims to analyse findings in the light of this historic-legal framework.

As the use of traditional and complementary medicine (T&CM) medicine increases [24], it is assumed that regulation is important to ensure the safety of the public using T&CM products and services. The WHO has encouraged member countries to pursue implementing a system of regulation for T&CM [24]. South Africa started the process of regulation of CM in 1974 – the aim being to regulate and control CM practitioner numbers by enforcing registration and closing all training facilities for CM. This regulation has its roots in the early 1950’s when medical practitioners began to campaign against the growing number of CM practitioners [25]. In 1953 alternative therapies were declared to be unscientific and illegal by the Medical Association of South Africa and the medical code prohibited co-operation between allopathic and alternative practitioners [26]. The resultant effect of this regulation was to radically reduce the number of CM practitioners, especially naturopaths [8, 27].

Current legislation defines naturopathy, sets the educational standards for training, determines the SOP, sets conditions for registration and the code of ethical practice for naturopaths. Regulation of a profession might be seen as a legitimizing of the profession, thus giving practitioners more status [28], and providing the public with a clear definition of the profession, the treatment practices offered, as well as the assurance of the level of care which may be expected [28]. Participants in this research all understood that being registered allowed them to legally practice under the title of naturopath. Despite the challenges some participants experienced with registration, being registered affords naturopaths title protection [28] and allows naturopaths to enter private practice when they have the means to do so. Currently there are less than 100 registered naturopaths on the AHPCSA register [29]. The number of registered practitioners is small, given that naturopathy has been practised in South Africa for over 50 years [8]. It raises questions about the lack of growth of naturopathy in this country compared to the global growth of the profession [30].

Participants expressed concern that regulation does not serve the naturopathic profession, because it has an inhibitory effect on the profession. Naturopaths and other CM practitioners are prohibited from sharing practice rooms with allopathic practitioners registered with the HPCSA [23]. This regulation therefore excludes naturopaths from practising in integrative private practises as well as the public healthcare system and perpetuates the separation of allopathic medical practitioners from CM practitioners, contrary to the interest of patients. The WHO [24] advocates for universal health coverage, through integration of traditional and complementary medicine into health service delivery, as it allows patients to choose the form of healthcare service they most want to use. Wholistic, integrated care and patient choice may optimise health services and promote the best health outcomes for the population. It has been shown that collaborative practices facilitates improved care and as a result, improved health outcomes [31].

In South Africa the White Paper on the National Health Insurance Act (NHI), published in the Government Gazette No. 42598 of 2019 [32], sets out the proposed plan to ensure universal health coverage for all by 2030. It envisions multidisciplinary clinics being located within the community, where service providers will be private practitioners contracted by the state to work in the clinics. This could potentially create the opportunity for naturopaths to work in a primary healthcare (PHC) setting if regulations prohibiting AHPCSA registered CM practitioners from working in the same practice as HPCSA registered practitioners are withdrawn.

Naturopathy is growing globally, including on the African continent [30]. Lack of growth of naturopathy in South Africa goes against this trend. This research found that current regulation is one factor that hampers the growth of the naturopathic profession. Further investigation into the legislation and its effects on CM needs to be conducted.

A limitation of this study was the two different methods in which responses to the open-ended questions were obtained. However, due to the punitive manner in which the regulations were implemented in the 1970’s and 80’s [8], older naturopaths remain seemingly suspicious of anyone enquiring about naturopathy and what they practice. The repressive environment in which they trained and practiced has shaped their experiences and how they view an ‘outsider’ questioning them about their practice [8]. Due to the age of some participants, it appeared important to accommodate their requests. They have lived experience of all the changes in legislation and vast practice experience. This knowledge will be lost if it is not recorded, hence the importance of granting them a voice in this study.

## Conclusion

This research found that legislation affects all aspects of the naturopathic profession in South Africa. It prescribes the range of treatment practices used and determines who may use the title of naturopath. While naturopaths share a similar vision for a future role in the public healthcare system, this vision cannot be achieved under current legislation. In the light of the information provided by South African naturopaths, there is a need to review the current regulations, in view of the increased demand for CM and recommendations by the WHO for the integration of the different medical systems into the national public healthcare system. This could ensure universal and comprehensive healthcare is provided to the population.

Within the South African context this is the first research to be conducted about naturopathy as a diagnostic system of CM. Further research should include an examination of the changes required in the regulatory environment to promote naturopaths’ presence in the South African public healthcare system.

## Data Availability

The datasets used and analysed in the course of the study are not publically available in order to protect the identity of the participants. It is available from the corresponding author on reasonable request.

## Abbreviations

AHPCSA: Allied Health Professions Council of South Africa
CM: Complementary Medicine
COCHASA: Confederation of Complementary Health Association of South Africa
CRE: Council regulated examination
HPCSA: Health Professions Council of South Africa
NHI: National Health Insurance
PHC: Primary healthcare
SANA: South African Naturopathic Association
SOP: Scope of practice
T&CM: Traditional and Complementary Medicine
WHO: World Health Organisation
